# Nutritional and socio-economic factors associated with *Plasmodium falciparum *infection in children from Equatorial Guinea: results from a nationally representative survey

**DOI:** 10.1186/1475-2875-8-225

**Published:** 2009-10-08

**Authors:** Estefanía Custodio, Miguel Ángel Descalzo, Eduardo Villamor, Laura Molina, Ignacio Sánchez, Magdalena Lwanga, Cristina Bernis, Agustín Benito, Jesús Roche

**Affiliations:** 1 Centro Nacional de Medicina Tropical, Instituto de Salud Carlos III, Sinesio Delgado 6, Pab 13, Madrid 28029, Spain; 2 Research Unit, Fundación Española de Reumatología, Calle Marques del Duero, 5, 1, Madrid 28001, Spain; 3 Department of Environmental Health Sciences, University of Michigan, School of Public Health, Ann Arbor, MI 48109, USA; 4 Centro de Referencia para el Control de Endemias, Laboratorio de Referencia de Paludismo, Hospital Regional de Malabo, Equatorial Guinea; 5 Departamento de Biología, Universidad Autónoma de Madrid, Calle Darwin, 2 Campus Universitario de Cantoblanco, Madrid 28049, Spain

## Abstract

**Background:**

Malaria has traditionally been a major endemic disease in Equatorial Guinea. Although parasitaemia prevalence on the insular region has been substantially reduced by vector control in the past few years, the prevalence in the mainland remains over 50% in children younger than five years. The aim of this study is to investigate the risk factors for parasitaemia and treatment seeking behaviour for febrile illness at country level, in order to provide evidence that will reinforce the EG National Malaria Control Programme.

**Methods:**

The study was a cross-sectional survey of children 0 to 5 years old, using a multistaged, stratified, cluster-selected sample at the national level. It included a socio-demographic, health and dietary questionnaires, anthropometric measurements, and thick and thin blood smears to determine the *Plasmodium *infection. A multivariate logistic regression model was used to determine risk factors for parasitaemia, taking into account the cluster design.

**Results:**

The overall prevalence of parasitemia was 50.9%; it was higher in rural (58.8%) compared to urban areas (44.0%, p = 0.06). Age was positively associated with parasitemia (p < 0.0001). In rural areas, risk factors included longer distance to health facilities (p = 0.01) and a low proportion of households with access to protected water in the community (p = 0.02). Having had an episode of cough in the 15 days prior to the survey was inversely related to parasitemia (p = 0.04). In urban areas, the risk factors were stunting (p = 0.005), not having taken colostrum (p = 0.01), and that someone in the household slept under a bed net (p = 0.002); maternal antimalarial medication intake during pregnancy (p = 0.003) and the household socio-economic status (p = 0.0002) were negatively associated with parasitemia. Only 55% of children with fever were taken outside their homes for care, and treatment seeking behaviour differed substantially between rural and urban populations.

**Conclusion:**

Results suggest that a national programme to fight malaria in Equatorial Guinea should take into account the differences between rural and urban communities in relation to risk factors for parasitaemia and treatment seeking behaviour, integrate nutrition programmes, incorporate campaigns on the importance of early treatment, and target appropriately for bed nets to reach the under-fives.

## Background

Equatorial Guinea is a small West Central African country divided into a mainland region (Río Muni) and an insular one (Bioko as the main island). Traditionally, malaria has been a major endemic disease in the country [1,2], but new socioeconomic conditions are changing the situation and given the opportunity for elimination [3].

In the past, the country's economy was based on forestry and agriculture, but since oil was discovered in the mid 90's on the coastline, oil extraction has become the main economic activity of the country. The development of the oil industry has led to rapid urbanization and to a growing availability of resources to address important public health problems through public and private initiatives [4].

One of these initiatives has been the Bioko Island Malaria Control Project (BIMCP) mainly consisting of the indoor residual spraying (IRS) programme, that was established in 2004 to eliminate malaria infection in the island [5] and that is now being extended to the continental region [6]. The BIMCP has been followed by an important decline in the prevalence of the parasitaemia in the island in these past years (from 42% pre-intervention, to 18% in 2008) [7]. These are encouraging results towards a "sustained control" of the disease and give hope to a potential "elimination" in the island [3], although the varying results in the different sentinel sites suggest that factors other than vector control still play a role on transmission that should be identified before elimination can be achieved [8]. Moreover, the prevalence of parasitaemia in the mainland, which is home to about 80% of the country's population [9], remains over 50% in children under five years [7].

The identification of risk factors for parasitaemia at the country level can contribute to the elimination efforts in Bioko island. In addition, it will help improve the program as it is extended to the mainland region, where geographic and socio-demograhic conditions, including urbanization (27% in the mainland region versus 85% in Bioko island [9]), differ substantially from the island's.

Case management through early diagnosis and prompt treatment of all fever cases in malaria endemic areas is a cornerstone of the global strategy for malaria control [2]. Prompt diagnosis and timely malaria treatment within 24 hours after the onset of symptoms can reduce illness progression to severe stages, and therefore, decrease mortality. In order to design effective strategies to improve case management, it is important to have information on the treatment seeking behaviour of the population.

The aims of this study were to identify the social, behavioural, and nutritional factors that are associated with *Plasmodium falciparum *infection, and to describe treatment seeking behaviour for febrile illness in preschool children from Equatorial Guinea, using data from a national survey carried out in February 2004, before the BIMCP was implemented.

## Methods

### Study area and population

Equatorial Guinea is located in the Gulf of Guinea, with an overall area of 28,068 km^2 ^and a population of ≈ 500,000 inhabitants. The proportion of the population living in urban areas has increased from 27.1% in 1975 to 48.3% in 2003 [10]. Infant and under five mortality rates were 123/1,000 and 204/1,000 respectively; malaria accounted for 24% of the causes of death among children under five years of age in 2002 [11].

### Study design

A nationally-representative cross-sectional survey was conducted between February and March 2004. Sampling was carried out with the use of a multistaged, stratified cluster strategy. The strata were island and continental regions and rural and urban settings. Primary sampling units were the villages in the rural areas and the neighbourhoods in the urban settings. They were selected randomly and proportional to size according to the 1994 Population and Households Census [9]. Secondary sampling units were randomly selected households from an updated census from each cluster. Tertiary sampling units were the children. Only one child younger than five years of age per household was selected randomly, from a list with all the children < 5 years of age residing at home, resulting in a non self-weighted sample. The initial sample size was increased in prevision of missing data but replacement was not carried out at any of the sampling stages. The total number of children surveyed was 552.

### Data collection

A blood sample was obtained from participating children to determine the presence of malaria infection through microscopic examination of stained thick and thin films. Thin smears were fixed with methyl alcohol and think and thick smears stained with Giemsa. Films were examined with a 100× oil immersion optical microscopy. *Plasmodium *infection was defined as the presence of any asexual forms on thick or thin blood films. An absence of malaria parasites was reported when 500 leucocytes were counted and no parasite had been observed in the corresponding fields examined. Each sample was studied by two qualified laboratory technicians and a third technician was called in when there was a discrepancy in the result. A curative dose of sulphadoxine-pyrimethamine was given to all the children taking part in the study.

All children were measured and weighted according to standard WHO procedures by the same trained nutritionist [12]. Age was calculated from the reported date of birth and when the date of birth was not known (5.4% of the sample) age in months as reported by the care provider was registered.

The children's care providers were interviewed by trained local personnel, using a standardized questionnaire that included questions on demographics, household characteristics, child health and feeding practices, fever treatment-seeking behaviour and malaria prevention behaviours. The questionnaires had been previously translated into the main local language, Fang; and the option was given to the care provider to be interviewed in Fang or Spanish, which is one of the official languages in the country. Additional details on the sampling techniques and the data collection process have been described elsewhere [13].

### Statistical analysis

The primary outcome of interest was *Plasmodium *parasitaemia while a secondary outcome was the presence of fever during the two weeks prior to the survey.

Stunting, underweight, and wasting were defined as height-for-age, weight-for-age and weight-for-height Z-scores < -2 SD, respectively, according to the 2006 WHO Growth Standards [14].

Socio-economic variables were analysed using a socio-economic status (SES) index created by principal component analysis [15]. SES was estimated from several household characteristics and assets variables. According to the index, each household was assigned to tertile categories labelled as low, middle, and high.

Multivariate analysis to examine the socio-economic, nutritional and dietary predictors of *Plasmodium *infection indicators were carried out using logistic regression models for rural and urban strata separately and adjusting for potential confounding variables that were significant in the univariate analysis. Multivariate models included all variables for which adjusted estimates are presented. Data were weighted according to the selection probabilities and analysed with the complex samples procedures of SAS software [16], that take into account the clustering of the sample.

P values ≤ 0.05 were considered to be statistically significant.

### Informed consent

The national survey was approved by the Ministry of Health of Equatorial Guinea. The village and neighbourhood representatives were informed by an official letter from the Ministry of Health of the day of the visit and the scope of the study, and oral informed consent was obtained from all the children's parents or primary care providers.

## Results

The overall prevalence of parasitaemia was 50.9% and virtually all infections were by *P. falciparum *(95.4%); among children with positive slides, 8.7% were co-infected with another species and 4.6% were infected with *P. malariae *and/or *P. ovale*. Prevalence was higher in rural areas (58.8%) compared to urban areas (44.0%, p = 0.06).

Table 1 shows the results of the multivariate logistic regression for *P. falciparum *infection for urban and rural strata separately. Age was positively associated with *P. falciparum *infection in both urban and rural areas [odds ratios (95% CI): 7.8 (3.4, 17.9), 13.9 (3.1, 62.6), respectively]. In rural areas, distance to the closest health facility was positively associated with infection [odds ratios (95% CI): 2.9 (1.3, 6.0)], whereas the proportion of households with protected water, and having had an episode of cough in the previous 15 days were inversely associated with it, [odds ratios (95% CI): 0.3 (0.1, 0.8), 0.4 (0.2, 0.97), respectively].

**Table 1:** Factors associated with *Plasmodium *infection in children below 5 years old in Equatorial Guinea for rural and urban populations

		**RURAL**					**URBAN**					
		
		*Plasmodium *infection*						*Plasmodium *infection*				
		
**Factor**	**N (%)†**	**n (%)**	**se**	**Unadj. P**	**Adjusted Odds ratio (95% CI)**	**Adj. P**	**N (%)†**	**n (%)**	**se**	**Unadj. P**	**Adjusted Odds ratio (95% Cl)‡**	**Adj. P**
**Child age**												
0-5 months	37(11,4)	11(22.4)	8.5		Reference		28 (11.4)	8(25.9)	11.0		Reference	
6-11 months	53(16.8)	26 (49.1)	8.6		2.54 (0.75,8.67)		41 (17.5)	11 (42.4)	17.5		6.42(1.08, 38.06)	
12-23 months	74 (25,6)	44 (59.9)	6.4		3.81(1.30,11.17)		58(23.5)	18(30)	6.0		3.50(0.82,14.87)	
24-59 months	144 (46,2)	99 (70.5)	5.8		7.82(3.42,17.92)		113(47.6)	60 (55.7)	6.3		13.94(3.10,62.62)	
*p *for trend§				0.0008		<.0001				0.07		0.004
**Sex**												
female	147 (43.9)	85 (60.3)	7.1		Reference		118(47.9)	43 (45.8)	7.8		Reference	
male	163 (56.1)	96 (57.6)	4.8	0.70	0.85(0.48,1.51)	0.28	124(52.0)	54(41.7)	6.4	0.60	1.49(0.75,2.97)	0.25
**Distance to closest health facility in km**												
less than 0.5 kms	184 (55.6)	102 (51.8)	6.7		Reference		149 (58.8)	57 (44.3)	9.1	...		
between 0.5 and 10 kms	91 (27.5)	50 (58.1)	7.1		1.19(0.47,2.89)		93(41.2)	40 (42.7)	7.2	0.86	...	
10 kms or more	35(16.9)	29 (82.7)	4.4		2.86(1.35, 6.03)						...	
*p *for trend§				0.005		0.01						
**% of households with access to protected water in the community**												
less than 30%	120 (42.1)	72 (66.2)	7.1		Reference		34(18.7)	19(53.7)	9		...	
between 30 and 60%	117 (38.3)	79 (64.3)	6.5		1.24(0.52,2.98)		30(10.2)	17(67.1)	15.4		...	
more than 60%	73(19.6)	30 (31.7)	7.6		0.29(0.11,0.77)		178(71.1)	61 (37.9)	7.5	0.63	...	
*p *for trend§				0.03		0.02						
**Episode of cough in the 15 days prior to the survey**												
No	114 (37.6)	75 (68.0)	6.8		Reference		111 (45.5)	43 (40.8)	7,4		...	
Yes	193 (62.4)	105 (53.1)	5.4	0.07	0.43(0.20,0,97)	0.04	125(54.5)	52 (46.8)	7,6	0.49	...	
**Stunting**												
No	183 (58.3)	103 (55.9)	5.6		...		170(71.5)	61 (38.9)	6.9		Reference	
Yes	118 (41.7)	73 (62.3)	6.6	0.40	...		57(28.5)	32 (53.6)	8.0	0.06	3.07(1.40,6.73)	0.005
**Colostrum**												
No	60(19.9)	38 (67.7)	7.3		...		45(19.0)	26 (65.8)	8.3		Reference	
Yes	235 (80.1)	135 (56.4)	5.7	0.18	...		170(81.0)	62 (39.8)	7.0	0.004	0.24(0.08,0.71)	0.01
**Mother took antimalaric drugs during pregnancy**												
No	126 (41.5)	74 (61.4)	4.6		...		84 (38.5)	40(53.1)	9.2		Reference	
Yes	184 (58.5)	107 (56.9)	6.2	0.39	...		157(61.5)	57 (37.5)	5.5	0.06	0.25 (0.098, 0.62)	0.003
**Someone in the house sleeps with bednet**												
No	189 (59.5)	107 (59.7)	5.5		...		87 (35.2)	33 (36.7)	6.4		Reference	
Yes	127 (40.5)	74 (57.8)	5.5	0.74	...		153(64.8)	64 (47.7)	7.1	0.14	3.05(1.52,6.14)	0.002
**Socio-economic level**												
low	101 (39.1)	59 (62.4)	6.5		...		81 (38.5)	38 (53.8)	8.3		Reference	
medium	109 (33.6)	63 (57.3)	6.5		...		79(31.7)	39(51.8)	8.1		0.97(0.29,3.25)	
high	100 (27.3)	59 (55.4)	7.8		...		82 (29.8)	20(21.6)	5.4	0.04	0.15(0.05,0.50)	
*p *for trend§				0.91								0.0002
Overall¶	308 (100)						240(100)					

*Plasmodium infection measured by means of thick and thin film.

† Frequencies are weighted and calculated according to the complex design taking into account clustering and stratification.

‡ Odds ratios obtained by multivariate logistic regresssion models for rural and urban strata. Models are adjusted for survey and clustering effects and include all

variables for which adjusted estimates are presented.

§ *p *value for the predictor when introduced into the model as a continuous variable representing the ordinal categories, adjusted for all other covariates in the multivariate model

¶ Totals may be less than 310 or 240 respectively, because of missing data.

... Not included on the multivariate model

In the urban settings; stunting, and the fact that someone in the house slept under a bed net were each positively related with *P. falciparum *infection in the child [odds ratios (95% CI): 3.1 (1.4, 6.7), 3.0 (1.5, 6.1), respectively]. On the other hand, children who had taken colostrum, those whose mothers had taken anti-malarial drugs during pregnancy and those from higher SES families had lower prevalence of *P. falciparum *infection [odds ratios (95% CI): 0.2 (0.1, 0.7), 0.2 (0.1, 0.6), 0.1 (0.05, 0.5), respectively].

When introduced in the multivariate models, no significant association was found between *P. falciparum *infection and the sex of the child; wasting, underweight or other morbidity indicators of the child; age, ethnicity, or education of the mother; breastfeeding; immunization status; household owning a domestic animal or a garden; child sleeping under a bed net or other community indicators, such as proportion of households with access to electricity in the community or the community endowment index.

Table 2 presents the correlates of treatment-seeking behaviour for the children that referred to have had fever in the 15 days prior to the survey (N = 256) by rural and urban settings. Almost 50% didn't seek any treatment for the fever, and in rural areas, 20% sought care only after two days or more of illness. In relation to the place attended, 30% looked for help in some kind of health facility (mainly hospitals in urban settings and health centres in rural places). In the urban setting significantly more people have turned to alternative places (mainly private drug stores) compared to rural settings. Regarding treatment, 40% reported to have treated children with anti-malarial drugs (mainly chloroquine and quinine).

**Table 2:** Treatment-seeking behaviour for febrile episoded in pre-school children from Equatorial Guinea in 2004 (N = 256)

	**Total**		**Rural**		**Urban**		
	
**Characteristics**	Frequency*	**se**	Frequency*	**se**	Frequency*	**se**	**P**
**Looked for help in Health facility**							
Hospital, health centre, private clinic	30,5	4,1	36,5	6,2	24,7	5,3	0,21
**Looked for help in other places**							
Pharmacy, market, witch doctor, family and others	26,7	4,5	17	3,8	36,2	7,3	0,02
**Mean time to seeking first treatment after fever onset**							
Same day	22,8	3,9	20	3,7	25,6	6,8	
Next day	15,9	3	14,8	4,3	16,9	4,2	
After two or more days	15,4	2,6	20	3,1	11	4,3	0,15
Didn't seek treatment	45,3	4,6	45,2	4,6	46,4	6	
**Treated with antimalaric drugs**	41,8	3,6	44,2	4,3	39,5	5,6	0,60
**Other kind of treatment**	82,5	2,3	86,7	3	78,5	5,3	0,58

*Frequencies are weighted and calculated according to the complex design taking into account clustering and stratification.

## Discussion

The prevalence of malaria parasitaemia in this study was 50.9%. This is consistent with results from previous years when the estimates of community prevalence of infection in children younger than 10 years of age exceeded 50% [1]. Although recent studies show a decline on infection on Bioko island, to 18% in children 2-5 years, the prevalence or *P. falciparum *parasitaemia on the mainland region remains very high, 59% for children under 5 years [7]. The socio-demographic and nutritional factors associated with *P. falciparum *infection for the whole country differ substantially for rural and urban populations.

Increasing age was a risk factor for *P. falciparum *infection in both urban and rural areas. It has already been established that, during the first months of life, the risk of infection is lower because there is still a degree of immunity from the mother. The risk of infection first increases with age and starts decreasing when the individual himself reaches a degree of immunity due to repeated exposure to the parasite [17].

In the rural settings, results show that the characteristics of the community have an important role on the individual risk of infection, including distance to health facility and the proportion of households with access to protected water. In previous results of this same survey the absence of high quality health services in the community was a risk factor for under nutrition only in rural areas, whereas a low community endowment index was a risk factor for anaemia in these same areas. Both these variables were associated with the negative effects of the process of rapid urbanization in the deterioration of rural communities [13]. The fact that an episode of cough on the 15 days prior to the survey resulted protective to *P. falciparum *infection might be associated with the common practice in the African Region to give anti-malarials presumptively to all patients who present with fever [2].

Among urban children, risk factors for parasitaemia differ substantially from the ones found in rural areas. From the nutritional perspective, stunting and not having taken colostrum are positively associated with *P. falciparum *parasitaemia. The relation between under-nutrition and malaria has been controversial for many years, but recent meta-analyses suggest that under nutrition is an important underlying risk factor for infectious diseases in general [18] and for malaria in particular [19]. More specifically, it has been recently shown that severe stunting induces down-regulation of the overall anti *P. falciparum *IgG Ab response [20]. The fact that not having taken colostrum is positively associated with infection could be associated with the immunological properties already established for breast milk and colostrums [21,22], and more specifically with the presence of anti-malarial antibodies in breast milk from immune mothers [23]. Although there is no association with breastfeeding, this could be due to lack of statistical power in the analyses, as non-breastfeeding prevalences were very low, 3.6% and 6.8% for rural and urban populations, respectively [13]. The fact that colostrum appears as a risk factor only in the urban settings might be related to the changes in infant feeding practices detected for this population in previous studies [13]. However, there is little information in the literature regarding this relation. Further research on the possible protective properties of colostrum against infection by *Plasmodium *is warranted.

The usage of mosquito nets is more frequent among the urban population than among the rural (64.8% of the urban households reported someone sleeping under a bed net at the time of the survey compared to the 40.5% of the rural households). However, no significant association was found between infection and children sleeping under a bed net in any of the two settings. This result does not coincide with the analyses of only the Bioko island population for this same survey (without rural/urban stratification) [24], and goes against the established evidence of the experimental studies that show a protective effect for the children sleeping under a bed net [25,26]. It was also surprising to find a positive association of infection with the fact that someone in the household sleeps under bed net, as other studies have suggested that community-wide effects of insecticide treated nets (ITNs) and bed net density at household level have a significant protective effect on child mortality [27,28]. However, these studies highlight the importance of bed net being treated with insecticide for this effect to be shown, and that information was not collected in the present survey.

Furthermore, recent studies in the region point out that the individual protection that children receive from the bed net density of a household is because children sleep with their parents, as bed net allocation is not prioritised to children less than five years of age but to the individual adults [29,30]. The hypothesis is that if someone in the household sleeps under a bed net it is because of a higher risk perception of infection due to environmental circumstances (mosquito density, wall construction material and/or conditions, etc.) that would place the unprotected child to risk.

The protective association of maternal intake of anti-malarial medication during pregnancy can be related to the shown effects on reducing placental malaria, maternal anaemia and low birth weight in other African countries [31-33], factors that can predispose the child to infection later in life.

The relation between poverty and malaria has been widely described [34] and specifically for urban areas in other African countries undergoing a socio-demographic transition, where rapid and unplanned urban growth creates suitable condition for malaria transmission. People who migrate from rural areas generally settle in poorly constructed houses in densely populated and underdeveloped peri-urban areas and bring along their traditional rural practices that may favour mosquito breeding [35,36].

Only 55% of the febrile children were taken outside their homes for care. In urban areas more children were taken to private drug stores than to health facilities and in rural areas around one fifth sought care only after two days or more of illness.

To reduce mortality from febrile illnesses and to prevent transmission, sick children not only need to get efficacious and appropriate drugs, but also need to get them in time; which WHO recommends being within 24 hours from illness onset [2]. The delay in care-seeking in the rural areas has been associated in other studies in Africa with low household socio-economic status, [37,38], and with geographical proximity to the provider [39,40]. In the rural areas in Equatorial Guinea the more accessible providers for caretakers are the government health posts, which are poorly provided and sometimes within more than 24 hours of reach. In urban areas there is a wider offer of private drug stores that can be closer to the community than health facilities. The number of children that referred to have been treated with anti-malarial drugs is consistent with the reported 38% of all African children in 2006 [2], and the fact that the main anti-malarial given was chloroquine instead of the more recommended artemisinin-based combination therapy is likely to have changed now, as the National Protocol was revised on 2006 [41] based on previously reported chloroquine resistances in the country [42].

## Conclusion

Results suggest that a national programme to fight malaria in Equatorial Guinea should a) integrate nutrition programmes specially designed for rural and urban populations, b) incorporate information, communication and education (ICE) campaigns on the importance of correct diagnosis and early treatment in rural and urban communities, c) include the reinforcement of the health system and improve the access to health facilities in rural areas or the provision of early diagnoses and treatment made through a programme of home-based management of malaria and d) design an appropriate targeting for bed nets to reach the under-fives and consider the importance of distributing long-lasting insecticide nets (LLINS).

## Conflict of interests

The authors declare that they have no competing interests.

## Authors' contributions

EC was involved in survey design and data collection, performed the statistical analysis and interpretation of the data and drafted the manuscript. MAD was involved in the survey design, the interpretation of the statistical analysis and helped to draft the manuscript. EV was involved in the performance and interpretation of the statistical analysis and revised critically the manuscript. LM was involved in the data collection and helped to draft the manuscript. IS participated in the survey design, the data collection and helped draft the manuscript. ML was involved in the data collection and helped to draft the manuscript. CB participated in the survey design, the interpretation of statistical analysis and helped draft the manuscript. AB participated in the survey design, the interpretation of statistical analysis and helped draft the manuscript. JR participated in the survey design and data collection, the performance and interpretation of statistical analysis and coordinated the draft of the manuscript. All authors read and approved the final manuscript.
